# The conservation value of admixed phenotypes in a critically endangered species complex

**DOI:** 10.1038/s41598-020-72428-2

**Published:** 2020-09-23

**Authors:** Keren R. Sadanandan, Gabriel W. Low, Sheeraja Sridharan, Chyi Yin Gwee, Elize Y. X. Ng, Pramana Yuda, Dewi M. Prawiradilaga, Jessica G. H. Lee, Anaïs Tritto, Frank E. Rheindt

**Affiliations:** 1 Department of Biological Sciences, National University of Singapore, Singapore, 117558 Singapore; 2 Max Planck Institute for Ornithology, 82319 Seewiesen, Germany; 3 School of Biological Sciences, Monash University, Clayton, VIC 3800 Australia; 4 Department of Biological Sciences, University of Alberta, 116 St & 85 Ave, Edmonton, AB T6G 2R3 Canada; 5 Universitas Atma Jaya, Jl. Babarsari 44, Janti, Caturtunggal, Kec. Depok, Kabupaten Sleman, Daerah Istimewa Yogyakarta 55281 Indonesia; 6 Division of Zoology, Research Center for Biology, Indonesian Institute of Sciences (LIPI), Jalan Raya Jakarta Bogor KM 46, Cibinong Science Center, Cibinong, 16911 Indonesia; 7 Wildlife Reserves Singapore, 80 Mandai Lake Road, Singapore, 729826 Singapore

**Keywords:** Evolution, Evolutionary genetics, Population genetics, Speciation, Taxonomy

## Abstract

In today’s environmental crisis, conservationists are increasingly confronted with terminally endangered species whose last few surviving populations may be affected by allelic introgression from closely related species. Yet there is a worrying lack of evidence-based recommendations and solutions for this emerging problem. We analyzed genome-wide DNA markers and plumage variability in a critically endangered insular songbird, the Black-winged Myna (BWM, *Acridotheres melanopterus*). This species is highly threatened by the illegal wildlife trade, with its wild population numbering in the low hundreds, and its continued survival urgently depending on *ex-situ* breeding. Its three subspecies occur along a geographic gradient of melanism and are variably interpreted as three species. However, our integrative approach revealed that melanism poorly reflects the pattern of limited genomic differentiation across BWM subspecies. We also uncovered allelic introgression into the most melanistic subspecies, *tertius*, from the all-black congeneric Javan Myna (*A. javanicus*), which is native to the same islands. Based on our results, we recommend the establishment of three separate breeding programs to maintain subspecific traits that may confer local adaptation, but with the option of occasional cross-breeding between insurance populations in order to boost genetic diversity and increase overall viability prospects of each breeding program. Our results underscore the importance of evidence-based integrative approaches when determining appropriate conservation units. Given the rapid increase of terminally endangered organisms in need of *ex-situ* conservation, this study provides an important blueprint for similar programs dealing with phenotypically variable species.

## Introduction

Our planet is heading into an extinction crisis, and conservation resources are finite^1,2^. This quandary has led to conflicts among conservationists, some of whom call for taxonomic practices that err on the side of elevating distinct populations to species level, whereas others fear ‘taxonomic inflation’ is diluting the value of conservation efforts^3^. In the context of the question of how much biological diversity we can afford to save, the taxonomic problem has featured prominently in conservation debates^4,5^, and has generated a panoply of partly overlapping, partly conflicting streams of action, such as the consideration of phylogenetic diversity in conservation^6^, extensions of environmental legislation to subspecies level in various jurisdictions^7^, and the formulation of multiple mutually non-overlapping definitions of ‘conservation units’^8^.

Biological diversity is typically generated slowly and cumulatively through evolutionary processes, which is why most conservationists agree that deeply diverged or distinct lineages deserve preferential conservation attention as compared to shallow lineages^9^. However, the Next Generation Sequencing (NGS) revolution of the last 10 years has increasingly shown that some species have taken considerable ‘short-cuts’ in the generation of substantial phenotypic differentiation, by-passing hundreds of thousands to millions of years of independent evolution by appropriating novel traits through horizontal gene transfer and genetic introgression^10–13^. This transfer of traits is now well-documented across a wide variety of model and non-model organisms^14–20^. Such phenotypic appropriation has occurred even in some populations of modern humans (*Homo sapiens*) which are known to carry traits conferred to them through genetic introgression from ancient, now-extinct hominine lineages^21,22^. Genetic introgression can sometimes lead to great phenotypic differences between those members of a species affected by interspecies admixture and those that are not. The significance of these processes for conservation, however, has so far been widely ignored. Should special conservation action be extended to phenotypically-different populations of an endangered species, even in cases where this may be a product of introgression rather than deeper evolutionary divergence?

**Figure 1:**
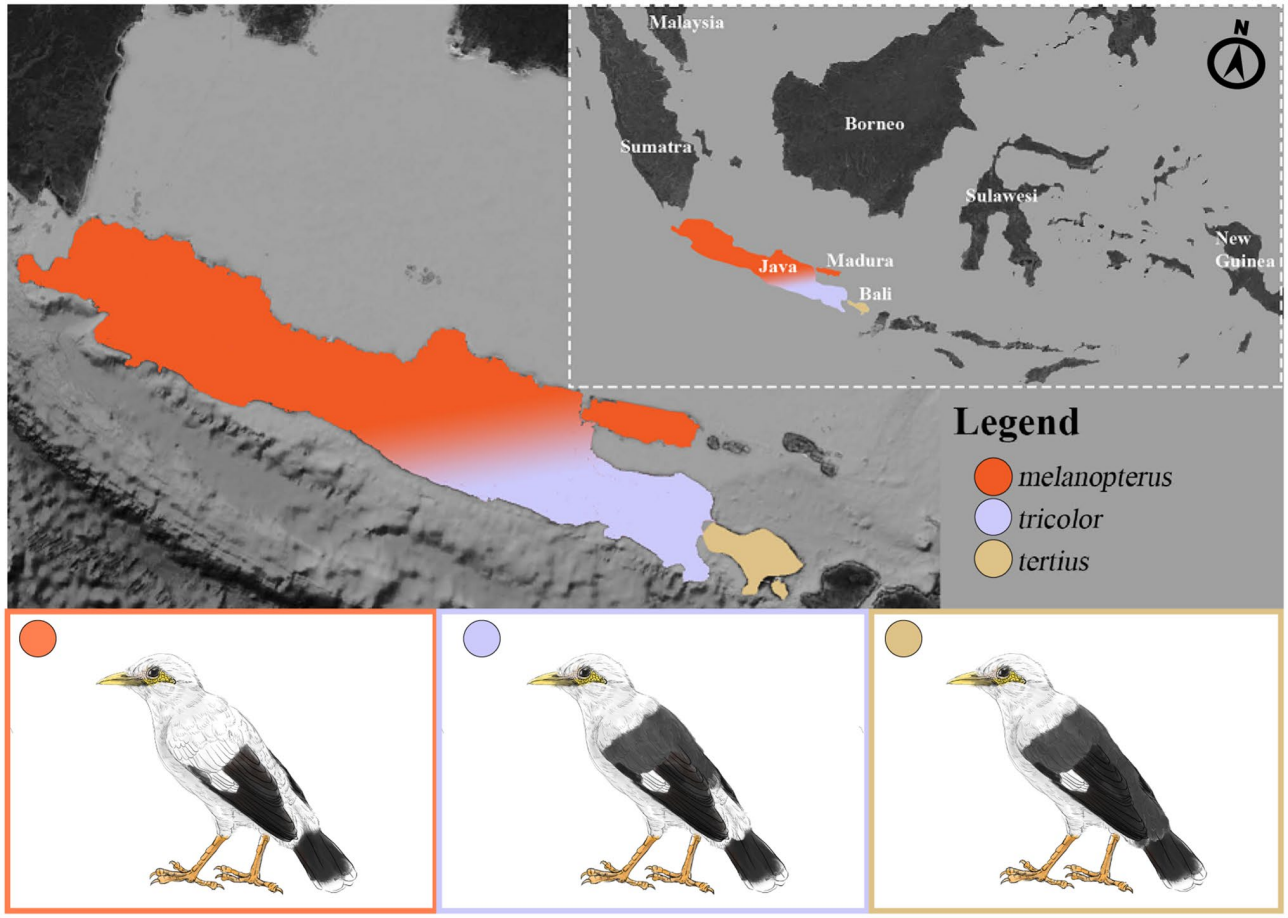
Map of Java and Bali showing the distribution of the three Black-winged Myna *Acridotheres melanopterus* subspecies (*melanopterus*, *tricolor* and *tertius*). Inset shows the location of Java and Bali within the Indonesian Archipelago. Myna drawings are by Yifan Pei. Map modified from https://maps-for-free.com/ (OpenStreetMap contributors) using Adobe Photoshop v.21.2 (https://www.adobe.com/products/photoshop.html).

To shed light on this question, we used population-genomic methods to analyze differentiation within the Black-winged Myna (*Acridotheres melanopterus*; BWM), a Critically Endangered songbird endemic to Java and Bali that is almost extinct in the wild due to illicit poaching pressure^23^. The species has been identified as a focal target requiring urgent conservation attention by IUCN’s Asian Songbird Trade Specialist Group and the Asian Species Action Partnership. BWMs have traditionally been divided into three distinctly-colored subspecies: (1) nominate *melanopterus* from West and Central Java, extinct in the wild as of 2018 except for a small feral population in a commercial wildlife park (T. Sumampau, pers. comm.), which has an almost entirely white body coloration apart from its black remiges and uppertail coverts; (2) East Javan *tricolor,* thought to number a few dozen individuals in the wild in two national parks, which is characterized by a greyish-black mantle with a distinctive white rump, and (3) Balinese *tertius*, with ~ 200 wild individuals thought to survive in Bali Barat National Park, which is the darkest subspecies with a dark-grey to black plumage coloration over its entire mantle and rump^24–29^ (Fig. 1).

With poaching pressure unrelenting across Java and Bali, conservation breeding has been strongly recommended as one of the main strategies in preventing the BWM’s extinction^25,30–32^. However, such efforts have been hampered greatly by taxonomic uncertainty^25^. The IUCN itself has followed a recent taxonomic re-assessment that elevates the three subspecies of the BWM to species level^33^ based on variation in plumage and biometrics^34^. This treatment has been controversial within the IUCN’s Asian Songbird Trade Specialist Group^32^, and has not been followed by any of the other major global or regional avian taxonomic authorities^26,35–37^. (We do not follow this treatment in this study either and predominantly refer to the three entities as ‘taxa’). This taxonomic uncertainty has generated confusion among conservation breeders as to whether it is appropriate to cross-breed the three taxa or not. To make matters worse, only two of the three taxa are held by captive breeding programs, and the levels of genomic differentiation between them are unknown.

Genomic tools play an increasingly crucial role in conservation management^38,39^, not only by inferring evolutionary patterns of divergence, identifying unique lineages and fine-scale patterns in population structure^40–42^ or revealing the hidden impact of illegal wildlife trade^43,44^, but also by being capable of identifying kinship groups and assessing the genetic viability of individuals within breeding populations^45,46^. In the case of the BWM complex, genomic tools allow for an assessment of whether to maintain separate breeding programs for each taxon, as suggested by the taxonomic arrangement recognized by the IUCN, or a single combined breeding program for the entire species complex. The former approach risks genetic inbreeding due to bottlenecks in the captive populations, while the latter approach risks the loss of evolutionarily unique lineages due to hybridization, so the consequences of incorrect decision-making will unavoidably impact the genetic makeup of resultant insurance populations generations into the future. It has become clear that a rigorous *ex-situ* breeding strategy for the conservation of the BWM must be evidence-based, and should preferably include an assessment of both genomic and phenotypic levels of differentiation among subspecies, along with an evaluation whether some of the phenotypic differences may have been generated by processes such as introgression.

In this study, we use thousands of genome-wide markers from 85 captive individuals across the morphological spectrum in the BWM complex to evaluate whether the phenotypic differences between the two geographically terminal taxa available in *ex*-*situ* breeding programs (*melanopterus* and *tertius*) are reflective of deep genomic variation. We also include single-gene data from the remaining taxon *tricolor* to allow for an estimation of divergence levels among all three extant taxa. Last but not least, we discuss the implications arising from the detection of differential secondary gene flow between various BWM populations and a sympatric congener, the Javan Myna (*Acridotheres javanicus*). This assessment allows us to provide recommendations for immediate and urgent conservation action.

## Results

### Sampling regime and plumage analysis

**Figure 2:**
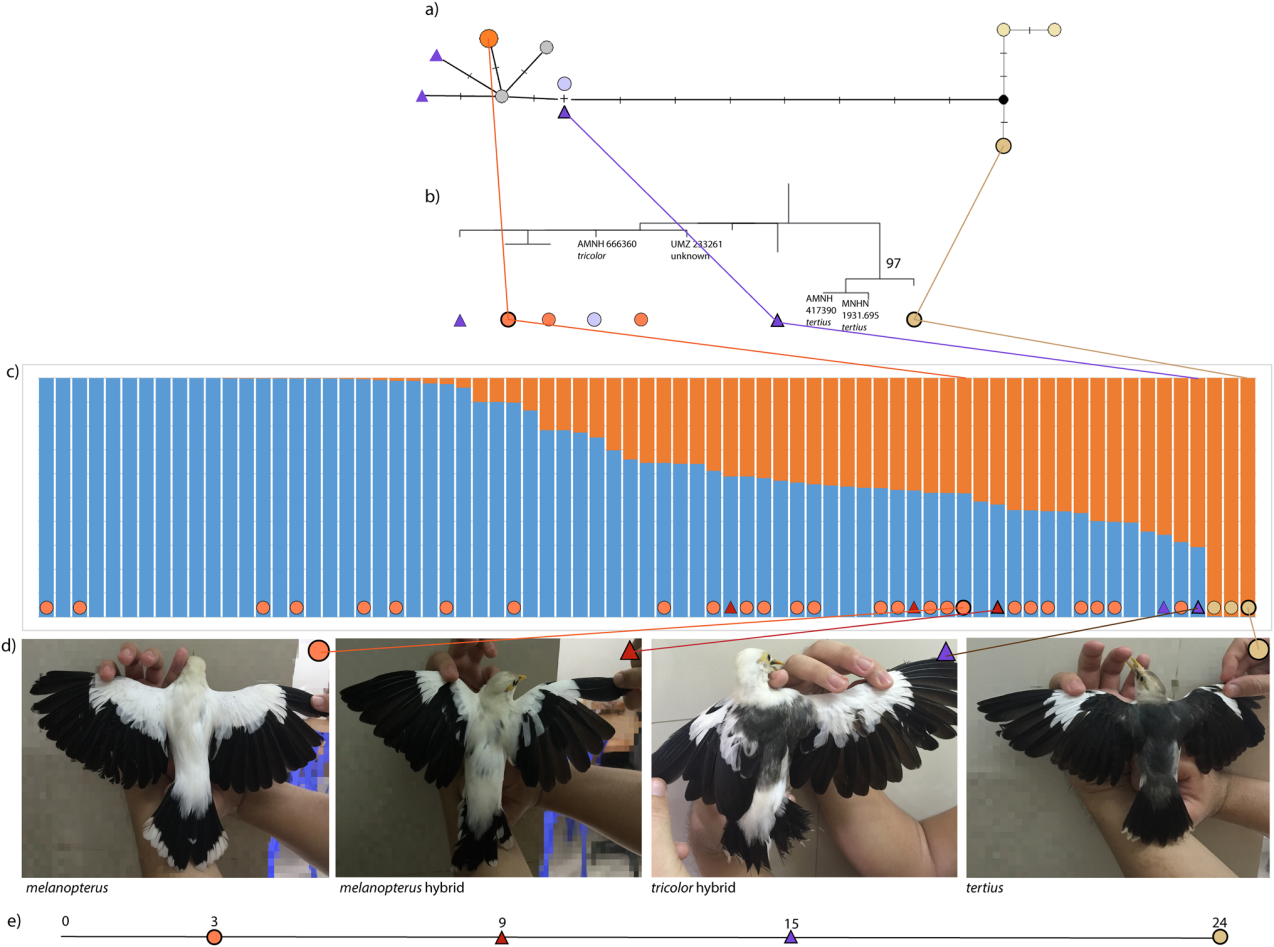
Subspecific variation within Black-winged Mynas (BWM). Mitochondrial *ND2* gene variation of a subset of samples representative of each taxon was visualized using (**a**) a median-joining haplotype network using a trimmed 902 bp alignment, and (**b**) a phylogenetic tree based on a maximum likelihood (ML) topology using an untrimmed alignment of 976 bp. Hatchmarks on the network correspond to single nucleotide changes. The four labelled tips on the ML tree are sequences obtained from GenBank. The remaining samples were sequenced for this study and are marked with a symbol connecting them to their position on the haplotype network and STRUCTURE plot (if applicable). The ML tree was rooted using a Common Hill Myna (*Gracula religiosa*). (**c**) Bayesian clustering analysis of 73 BWMs (filtered to exclude any first-order kin) in STRUCTURE with 7,229 genome-wide SNPs. (**d**) Upperparts coloration of four representative BWM individuals, including the two terminal subspecies (*melanopterus* and *tertius*) as well as two morphological hybrids (*melanopterus*-like hybrid and *tricolor-*like hybrid), relative to our plumage scoring scheme. (**e**) A color score of 0 corresponds to the lightest-backed individuals (*melanopterus*) and a score of 24 corresponds to the darkest individuals (*tertius*). Colored symbols represent four classes of morphological identity assigned to samples according to photos at the bottom. Samples without a morphological identity lacked photos, but can be assumed to be of the pure *melanopterus* phenotype (see “Results”).

We scored melanistic characteristics of a subset of BWMs, sourced from two conservation breeding facilities, that were classified as either *melanopterus*, *tertius* or hybrids based on plumage and studbook records. Our phenotypic score range extended from 0 to 24 along a cline of increasing melanism (Fig. 2). We did not include individuals identified a priori as *tricolor* in our analysis, as this nearly extinct taxon is not currently kept in any conservation-breeding facility (see “Introduction”). However, of the 39 individuals scored, five *melanopterus* individuals displayed intermediate phenotypes with scores between 8 and 21 (‘hybrid’ phenotype), with two in particular exhibiting traits typically diagnostic of *tricolor* (grey back and white rump) (Fig. 2). We therefore distinguished between overall whiter ‘*melanopterus*-like’ and overall darker ‘*tricolor*-like’ hybrids. Of the remaining individuals, 28 had a cumulative colour score of 0–6 (‘typical *melanopterus’* phenotype) and six individuals had a score of 22–24 (‘typical *tertius’* phenotype). Birds that were not scored were classified as ‘Unknown’ in our subsequent genomic analysis—however, it should be noted that these ‘Unknown’ individuals were all listed as ‘*melanopterus*’ by their breeding facilities, and are thus assumed to fall within the pure *melanopterus* score range of 0–6.

### Genomic-wide data and its variability across Black-winged Mynas

We used different filtering options to generate four genomic datasets, with between 6,999 and 13,841 genome-wide single nucleotide polymorphisms (SNPs), from our double digest Restriction-Site Associated DNA sequencing (ddRADSeq) data. These datasets included: (1) all 85 individuals, (2) individuals with no first order kinship, (3) founder individuals identified by pedigree information from Cikananga Conservation Breeding Centre and any individuals with a kinship coefficient less than 0.25, and (4) a genus-level subset for introgression testing, which included congeneric Javan Mynas (*Acridotheres javanicus*) and Common Mynas (*Acridotheres tristis*) (Table 1).

**Table 1:** Summary of datasets generated during SNP-calling, with taxa incorporated, sample sizes and SNP harvests reported.

Name of dataset	Taxa included	Sample size (individuals)	SNP harvest
Dataset 1 (full dataset)	*A. m. melanopterus* *A. m. tertius* *A. m.* morpho-hybrids	85	6,999
Dataset 2 (No_kin)	*A. m. melanopterus* *A. m. tertius* *A. m.* morpho-hybrids	73	7,229
Dataset 3 (founders)	*A. m. melanopterus* *A. m. tertius* *A. m.* morpho-hybrids	28	7,983
Dataset 4 (Con-generic comparisons)	*A. m. melanopterus* *A. m. tertius* *A. m.* morpho-hybrids *A. javanicus* *A. tristis*	20	13,841

**Figure 3:**
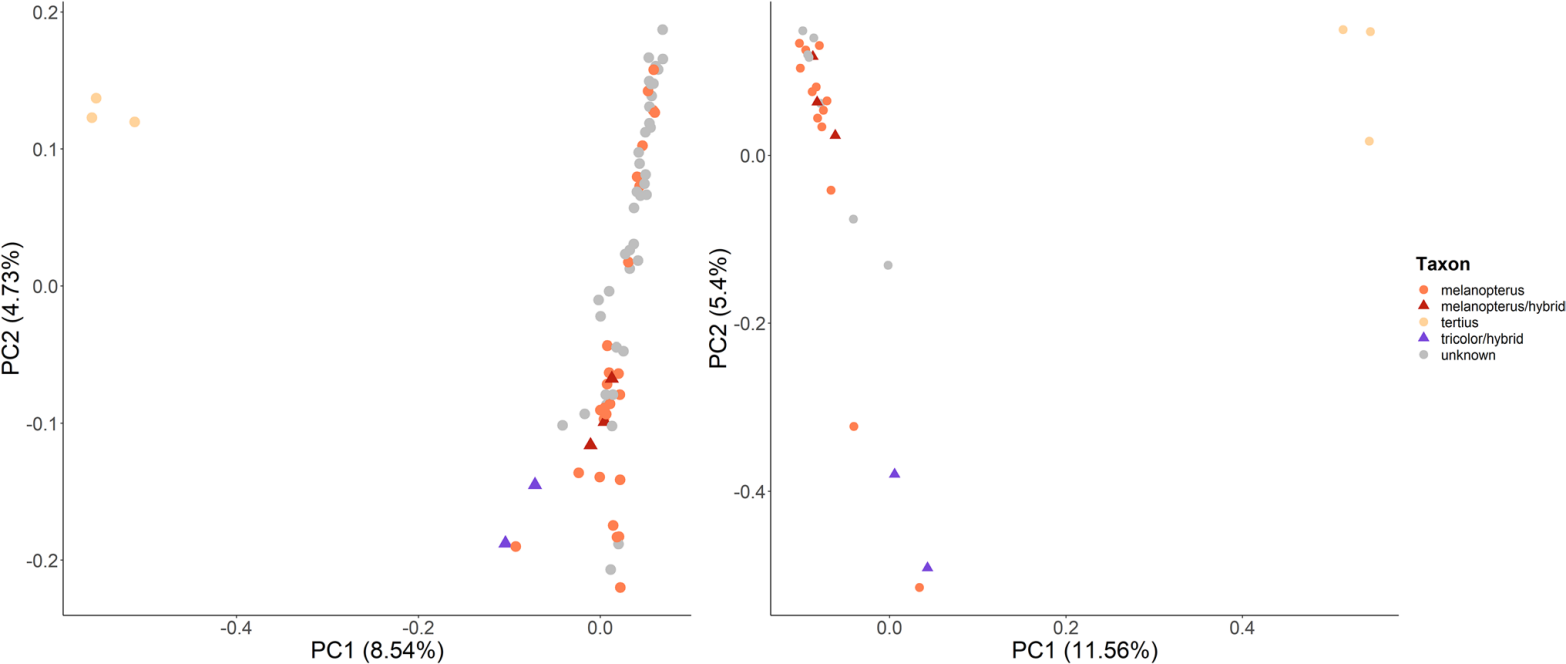
Population subdivision of Black-winged Myna samples by principal component analysis (PCA) for dataset 2 (left; comprising the larger captive dataset with kin filtered out) and dataset 3 (right; comprising the smaller dataset of only captive founders based on pedigree data). The PCA for dataset 2 was based on 7,229 SNPs and explained 13.27% of the total variation observed, whilst the PCA for dataset 3 was based on 7,983 SNPs and explained 16.96% of the variation observed.

On principal component analysis (PCA) plots, our sampled BWMs are roughly divided into two clusters separated along principal component PC1: one cluster containing west Javan *melanopterus* and morphological hybrids (including *melanopterus*-like hybrids and east Javan *tricolor*-like hybrids), and a second exclusively containing Balinese *tertius* individuals (Fig. 3). While *tertius* individuals formed a tight population cluster, *melanopterus* individuals were arranged in a continuum along PC2, and were widely interspersed with morphological hybrids. This arrangement held regardless of whether datasets 2 or 3 were used (Fig. 3).

STRUCTURE results mirrored the population-genomic arrangement in PCA. We inferred an optimal division into two population clusters^47^ (Fig. 2). This division identified *tertius* individuals as a unique population, whilst the *melanopterus* individuals and hybrids were distributed across a gradient with varying levels of shared ancestry with the *tertius* group (Fig. 2). Importantly, individuals identified as morphological hybrids (score range 8–21) emerged with variable percentages of *tertius* contributions, which often exceeded but sometimes remained below the level of genomic *tertius* contributions exhibited by some individuals identified as morphologically pure *melanopterus* (score range 0–6) (Fig. 2).

### Shallow single-gene divergences among clades

We were able to source multiple formerly published mitochondrial *ND2* gene sequences of BWMs from GenBank, including one from the rare East Javan taxon *tricolor*, which is not represented in any modern conservation-breeding facilities and therefore almost impossible to obtain. We compared these sequences with a subset of *ND2* sequences generated from our dataset. Our phylogenetic analysis revealed limited differentiation among all three subspecies (Fig. 2). The *tertius* samples emerged in one highly supported (bootstrap value 97) internal clade to the exclusion of all *melanopterus* samples, the single *tricolor* sample, and phenotypic hybrids. However, raw pairwise *ND2* divergence was very low among all samples (< 1.5%), especially when compared to mitochondrial divergences typically associated with sister species level in the bird barcoding literature (~ 2–3%)^48–50^.

We also detected no sequence variation in the *MC1R* gene across the subset of individuals we sequenced from our fresh dataset (including taxa *melanopterus*, *tertius* and morphological hybrids; Fig. S1), indicating that differences in mantle plumage colouration are not encoded in the *MC1R* gene, although they may be anchored in regulatory elements or other genes.

### Post-divergence genetic introgression

**Figure 4:**
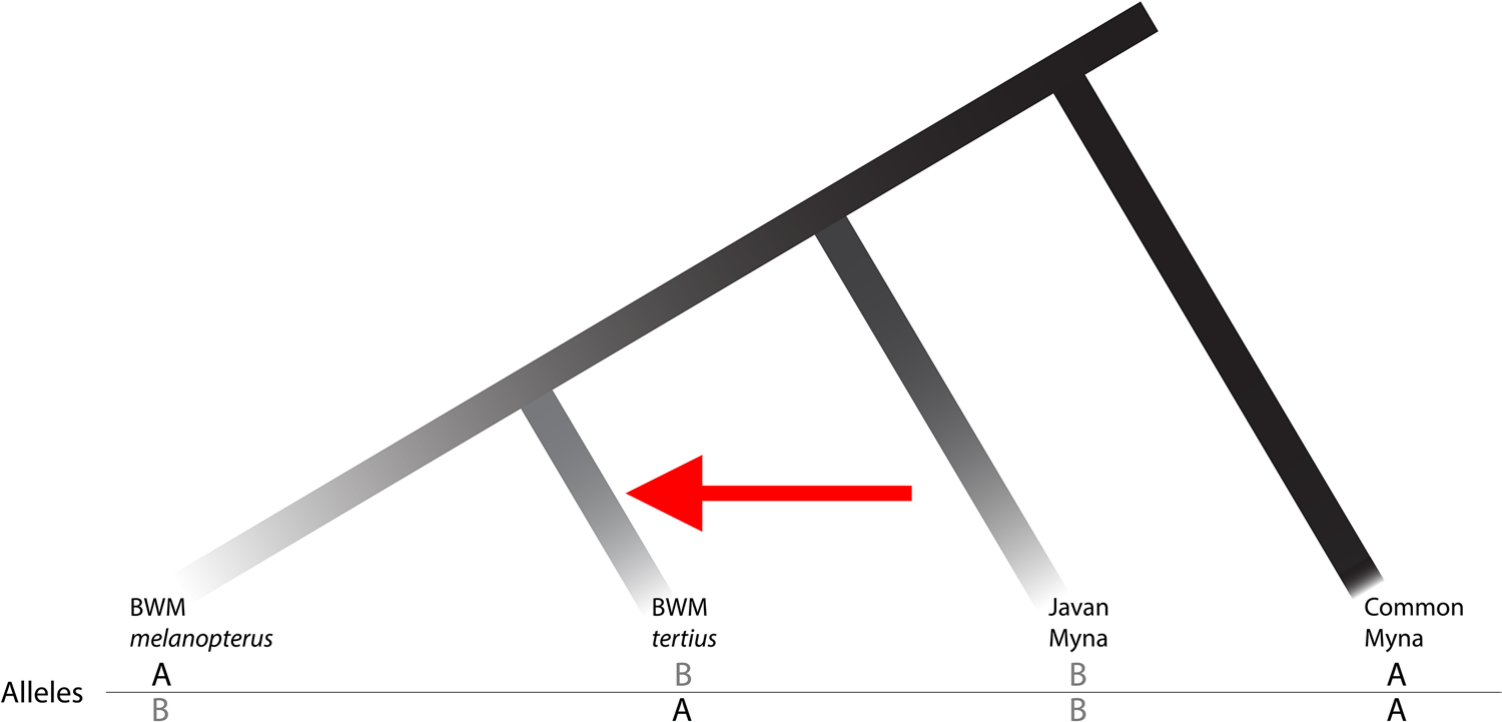
Four-taxon ABBA-BABA testing scheme for *Acridotheres* mynas. A four-taxon pectinate phylogeny shows two possible discordant gene tree patterns, ABBA and BABA, that typically occur in equal proportions under incomplete lineage sorting, with A and B denoting the ancestral and derived allele states respectively. Post-divergence gene flow (introgression) from lineage 3 to 2 generates additional instances of the ABBA pattern in this arrangement, leading to its preponderance over BABA arrangements. Branch tips have been labelled with tested taxa for clarity.

Population-based testing for secondary gene flow on dataset 4 (Table 1) revealed a significant level of genetic introgression from another species, the sympatric Javan Myna *A. javanicus*, into the Balinese population of the BWM (subspecies *tertius*) (D = 0.0373, *p* value = 0.0273, Table 2), relative to the West Javan subspecies *melanopterus*. Individual level testing showed significant introgression from Javan Myna into *tertius* individuals (BBP4 and BBP7) in eight of 28 *tertius-melanopterus* pair combinations tested in the arrangement depicted in Fig. 4 (Table 2). None of the pairwise combinations resulted in a signal of significant introgression from Javan Mynas into *melanopterus*. We estimated an admixture fraction *f*_G_ of 1.16% for the population level test, indicating that the latter percentage of the *tertius* genome is estimated to be secondarily derived from the Javan Myna through introgression.

**Table 2:** Results of introgression tests from Javan Myna (P3: *javanicus*) into *tertius* Black-winged Myna populations (P2), relative to *melanopterus* (P1).

P1	P2	P3	D-statistic	*p* value	*f*_G_
*melanopterus*	*tertius*	*javanicus*	0.0373	0.0273	0.0116
BWS605	BBP4	*javanicus*	0.0558	0.0468	0.0181
BWS612	BBP4	*javanicus*	0.0686	0.0108	0.0216
BWS615	BBP4	*javanicus*	0.0605	0.0255	0.0184
BWS625	BBP4	*javanicus*	0.0697	0.0038	0.0213
BWS626	BBP4	*javanicus*	0.0657	0.0094	0.0211
BWS605	BBP7	*javanicus*	0.0529	0.0425	0.0165
BWS612	BBP7	*javanicus*	0.0638	0.0126	0.0195
BWS615	BBP7	*javanicus*	0.0601	0.0272	0.0183

The first line reports the genome-wide D-statistic, *p* value, and admixture fraction (*f*_G_) of P2 for the population level test. Subsequent lines report these statistics for all tested pairwise combinations of *melanopterus* and *tertius* individuals that resulted in significant introgression (D > 0, *p* value < 0.05, 8 of 28 pairwise combinations tested). No combinations showed significant introgression from Javan Myna into *melanopterus* individuals relative to *tertius* individuals.

In order to further investigate allele sharing between Javan Mynas and BWMs, we ran both fineRADstructure and STRUCTURE on dataset 4 (Table 1) while excluding the Common Myna outgroup. When comparing Javan Mynas against the two geographically terminal subspecies of BWM (*melanopterus* and *tertius*), fineRADstructure indicated higher overall allelic sharing with *tertius* than with *melanopterus* (see Fig. S2). No allelic contribution from Javan into either of the two BWMs was immediately detectable in Structure analysis (Fig. S2), which suggests that the contributions of introgression found in the D-statistic tests only affect small parts of the genome, and are not due to any recent hybridization events over the last few generations, in agreement with our estimates of the admixture fraction *f*_G_.

## Discussion

The Black-winged Myna is representative of a growing global panel of terminally endangered species whose fate and continued survival crucially hinges on *ex-situ* breeding efforts coupled with intense management in the wild. Rampant illegal wildlife trade, especially in Asia, has led to a rapid proliferation of such terminally endangered species over the last five to ten years. This has included numerous parrots and songbirds, along with many non-avian animals^25,51–53^. Out of the three distinct subspecies of the Black-winged Myna, one (*melanopterus*) is practically extinct in the wild except for a small free-roaming flock inside a Javan wildlife park, while the other two subspecies each number < 200 individuals in the wild. Of equal worry, only one subspecies (*melanopterus*) was covered by an active conservation-breeding program numbering a few dozen individuals at the time of writing^25^, while the other two subspecies were either held in numbers too low for a viable breeding program (*tertius*) or were completely unrepresented in any of the world’s recognized conservation-breeding facilities and zoos (*tricolor*)^23,31^. All three subspecies of Black-winged Myna have been protected under the Indonesian Ministry of Environment & Forestry’s regulations since 2018.

Critically, the IUCN’s move to elevate the three subspecies to species level, in disagreement with the practice of most global and regional taxonomic authorities, has created a situation in which leaders of *ex-situ* programs are uncertain about best practices regarding the breeding of individuals bearing traits that reflect minor gene flow between subspecies. Specifically, the breeding program of *melanopterus* has been beset by a surprising incidence of individuals with isolated grey mantle feathers on otherwise white upperparts (Fig. 2), a phenotype often hitherto interpreted as a sign of past gene flow from *tricolor* or *tertius*. Although such individuals can potentially be carriers of important genetic diversity for the small captive population, they have generally been kept separate from the breeding program for fear of ‘phenotypic contamination’. However, the assumption that the so-called ‘purity’ of these individuals is in question has not been tested to date, and our study provides the first genomic evidence-based approach to assessing the biological importance of such admixture in guiding conservation efforts in this species complex.

### Melanism levels do not reflect deep genomic differentiation in Black-winged Mynas

In spite of a narrow geographic distribution restricted to Java and Bali, BWMs display an unusual amount of plumage differentiation that has led to recent taxonomic disagreements, prompting some authorities^28,29,33^, but not others^26,35,37^, to elevate all three subspecies to species level. However, our phylogenetic analysis of the mitochondrial *ND2* gene, including a single *tricolor* sequence from GenBank, demonstrated only shallow differentiation across all three taxa (< 1.5%; Fig. 2), well below that traditionally associated with species-level differentiation in birds using the same mitochondrial gene or other genes with similar evolutionary rates^48,49^.

Genome-wide analyses equally reject the notion that melanism reflects genomic differentiation at the species level in the BWM complex. Balinese *tertius* does form a genomic cluster distinct from Javan *melanopterus* individuals of varying degrees of melanism in principal component analyses (PCA; Fig. 3), whether we excluded kin or not. However, we observed a significant proportion of genomic variation (41–51%) shared between *tertius* and *melanopterus* in STRUCTURE modelling (Fig. S2). In combination with our results of low mitochondrial divergence among subspecies, this pattern of genomic differentiation is more consistent with *tertius* being distinct at the subspecies level rather than the species level.

More importantly, we found typical *melanopterus* individuals interspersed among hybrids of varying levels of melanism across a wide spread in genomic PCA space (Fig. 3). Morphological hybrids predominantly exhibited higher levels of shared ancestry with ‘typical’ *melanopterus* individuals than with ‘typical’ *tertius* ones in STRUCTURE analysis (Fig. 2). A lack of genomic divergence among Javan individuals with various degrees of melanism is perhaps unsurprising, given that historical records attest to a range overlap between *melanopterus* and *tricolor* along the Central and East Java border (Fig. 1), where individuals displaying intermediate morphotypes were collected in the wild^34^, evidencing historical gene flow between these two subspecies. Our results therefore indicate that East Javan *tricolor* may possibly fall on the opposite end of a smooth genomic cline with West Javan *melanopterus* if our ‘*tricolor*-like’ hybrids give any indication. We strongly advocate further genomic research involving known *tricolor* samples, preferably from historic museum specimens, to corroborate that the geographic and phenotypic cline between these two taxa was gradual.

### Impact of differential introgression on Black-winged Myna conservation

Genetic introgression entails the movement of alleles between otherwise well-established species as a consequence of rare hybridization events. Introgression is known to be pervasive in nature, especially birds^11^, and can lead to a quick appropriation of novel phenotypes, leading to unusual levels of morphological diversity in species that contain both pure populations and populations that have been recipients of alleles from another species. However, the significance of this process with regards to conservation has so far been widely ignored.

We discovered a signature of genetic introgression from the congeneric Javan Myna (*A. javanicus*) into the more melanistic *tertius* subspecies of BWM (Table 2), but not the lighter taxon *melanopterus*. Our results also indicate that the detected signal of introgression is unlikely to be recent (for example, as a result of anthropogenic hybridization in captivity). When investigating allele sharing between Javan Mynas and the two geographically terminal BWM subspecies (*melanopterus* and *tertius*), fineRADstructure indicated higher overall allelic sharing with *tertius* than with *melanopterus* (Fig. S2a, see darker yellow within black stippled line versus lighter yellow within black square), while no allelic contributions from Javan Myna into either of the two BWM subspecies was immediately detectable in STRUCTURE models (Fig. S2b). We estimate the introgression admixture fraction, *f*_G_, to be 1.16% in *tertius* (Table 2), indicating the percentage of its modern genome estimated to be derived from Javan Mynas through introgression. By comparison, the estimated proportion of Neanderthal ancestry in modern-day non-African humans is 1.3–2.7%^54^.

Our results are in good agreement with a hypothesis of differential introgression from all-black Javan Mynas into BWMs, with the darker *tertius* experiencing stronger introgression than the whiter *melanopterus*, if it occurred at all into the latter. The most plausible biogeographic explanation for such a pattern is Java and Bali’s aridity gradient^55^, which sees average annual precipitations gradually decrease eastwards from West Java’s former lowland rainforests to Bali’s dry monsoon woodland. Little is known about the differences in habitat preference of Javan Mynas and BWMs, although the former may always have occurred in more open situations, whereas the latter may have preferred denser woodland and forest^56^. It is conceivable that West Java would historically have allowed BWMs and Javan Mynas to segregate ecologically along clearer lines, while the drier, sparser habitat in East Java and Bali would have brought the two species into closer vicinity of each other, increasing chances of hybridization and thereby generating a smooth gradient of increasing melanism towards the east.

Even though BWMs and Javan Mynas have been known to be separated by shallow genetic differentiation for over a decade^57^, the possibility of introgression between them has never previously been raised in the literature because their markedly different plumage colorations make them appear to be unlikely hybridization partners. Our finding is the newest in a series that have shown that secondary gene flow leading to phenotypic change is more widespread than previously thought in birds^11,16,58–62^. While the admixture fraction estimated in *tertius* may appear small, melanistic plumage coloration in songbirds is thought to be determined by few genomic loci^63^. Consequently, a small number of introgressed alleles may well be responsible for their differentiated plumage. Our analysis of *MC1R*, a gene with known involvement in the melanism pathway in birds^63,64^, showed no variation across BWM individuals (Fig. S1), indicating that differences in melanism may be encoded in regulatory elements or other genes. Despite being unable to explicitly test Javan Myna introgression into *tricolor* due to the unavailability of known captive samples of this taxon, our results lead us to hypothesize that the degree of melanistic plumage traits in each BWM taxon is likely determined by its level of Javan Myna introgression. Future work including *tricolor* samples and whole-genome resequencing to facilitate local introgression scans and gene ontology enrichment analyses^65^ will have the analytical power to ascertain which introgressed elements in BWMs are responsible for the varying degrees of melanism among the three subspecies. In the meantime, the association of observed differences in melanism among BWM subspecies with differential introgression from the all-black Javan Myna raises important questions regarding the immediate conservation of these differently coloured forms.

### Recommendations for conservation of Black-winged Mynas

Extreme phenotypes that have been inherited through small-scale introgression can be easily mistaken as signals of species-level differentiation even if actual genomic differentiation between ‘pure’ and ‘introgressed’ populations is low. In a conservation context, such misdiagnoses can lead to poor practice. One of the most illustrative examples is the Coyote (*Canis latrans*): in certain areas of southeastern North America, Coyotes co-occur and hybridize with slightly larger-sized canids that have long been mistaken for an independent, highly endangered species, the ‘Red Wolf’ (*Canis rufus*)^66^. Recently, the Red Wolf was shown to be extremely similar to Coyotes genomically, albeit with a signature of excess allelic sharing with Grey Wolves (*Canis lupus*)^67^, suggesting that Red Wolves may not have existed in North America in pre-human times, but instead emerged as the result of a locally dying Grey Wolf population being assimilated with Coyotes. Regardless of ongoing disputes about the perceived conservation value of Red Wolves^68^, knowledge of the true evolutionary history of an introgressed lineage carries immense implications for conservation.

Our study showed that the two geographically and morphologically terminal forms of BWM, *melanopterus* in the west and darker *tertius* in the east, are characterized by a mtDNA divergence below the species level. Variation in levels of melanism, recently used to separate BWMs into three species, is not reflected by deep genomic differentiation, arguing in favour of the traditional taxonomic arrangement that unites all three forms as subspecies rather than species. Our demonstration of differential genetic introgression from a sympatric congener, the all-black Javan Myna, into the BWM’s darkest subspecies *tertius*, but not the whitest subspecies *melanopterus*, is likely linked to their corresponding levels of melanism. The main question for conservationists now is whether substantial resources should be invested into maintaining three *ex-situ* breeding programs, strictly separated from each other, to preserve morphological diversity that is likely caused by secondary introgression and not by deep genomic divergence.

In the present environmental and funding environment, we recommend aiming for three separate breeding sub-programs under the umbrella of a single species-wide program without a strict separation. One of these sub-programs already exists (subspecies *melanopterus*) whereas the other two urgently need to be established. The aim of this recommendation is to sample and preserve the whole range of morphological and genetic diversity that used to be found naturally in the range of this species, while retaining the option of cross-breeding subspecies. While we show that melanism is a poor indicator of overall genomic divergence in BWM, it is likely linked to local adaptations in each population that generate apparent phylogeographic breaks and resultant phenotypic clustering, as evidenced empirically in Western Barn Owls (*Tyto alba*) across Europe, as well as with simulations^69,70^. Melanistic phenotypes therefore likely provide good visual indicators of local adaptations, which may be crucial for the success of any possible wild reintroductions in the future^71,72^, while effectively hedging each captive population as insurance against the detrimental effects of genetic diversity loss over time, since individuals from each program can be drawn as donors for the genetic rescue of another^73^.

Finally, it is dangerous to exclude individuals from a breeding program based on slight deviations from the typical subspecific phenotype, as this removal deprives any small captive population of important genetic diversity, and is unwarranted considering that our results show that melanism is not a good indicator of taxon ‘purity’ in this complex. Exclusionary practices of individuals on the basis of isolated aberrant feathers do not mirror the former situation in the wild, where all three subspecies would have occurred along a cline, including *tertius* on Bali, an island that was connected to Java as little as ~ 11,000 years ago^74^.

To the best of our knowledge, our study on Black-winged Mynas is the first to deploy an integrative phenotypic and genomic approach to shed light on the conservation of a critically endangered species that is characterized by a genetic introgression gradient. Our approach has large comparative potential and can serve as a blueprint for other programs, given that more and more species’ survival will become dependent on *ex-situ* breeding efforts, including other species with phenotypic diversity caused by introgression. Our results underscore the importance of an evidence-based, integrative approach that contrasts phenotypic diversity with genomic differentiation and carefully weighs potential causes of morphological distinctness. One of the main limitations of our study is the use of only captive samples, which is likely mirrored by many other conservation-genomic datasets dealing with species that are nearly extinct in the wild, and a lack of genomic data from one of the three taxa, *tricolor*, again precipitated by the extreme rarity of this form. Future work on this species complex should strive to utilize data from wild samples, possibly in the form of historic museum specimens, in order to fully characterize the nature of divergence between the three forms, and to investigate fine-scale differences in the extent of introgression from Javan Mynas.

## Methods

### Sample collection

All samples used in this study were sourced from two collaborating conservation breeding facilities: Cikananga Conservation Breeding Center (West Java) and Bali Bird Park (Bali) (Material Transfer Agreement 2016-1858) (Table 3). Samples were collected by brachial venipuncture, whereby approximately 30 μl of blood was collected from each individual. Live animal sampling was approved by the Institutional Animal Care and Use Committee (IACUC) at the National University of Singapore. Cikananga Conservation Breeding Center additionally donated a further 30 tissue samples of previously perished individuals to be included in the analysis.

**Table 3:** List of *Acridotheres* myna samples used in this study, including morphological assignment and provenance.

Sample name	Morphological ID	Source	Photographic documentation	SRA accession number
BBP1˜	*A. m. tertius* hybrid	blood (live bird), Bali Bird Park	Y	Pending
BBP2*˜	*A. m. tricolor* hybrid	blood (live bird), Bali Bird Park	Y	Pending
BBP3*	*A. m. tricolor* hybrid	blood (live bird), Bali Bird Park	Y	Pending
BBP4	*A. m. tertius*	blood (live bird), Bali Bird Park	Y	Pending
BBP5	*A. m. tertius*	blood (live bird), Bali Bird Park	Y	Pending
BBP6*˜	*A. m. tertius*	blood (live bird), Bali Bird Park	Y	Pending
BBP7	*A. m. tertius*	blood (live bird), Bali Bird Park	Y	Pending
BBP8˜	*A. m. tertius*	blood (live bird), Bali Bird Park	Y	Pending
BBP9	*A. m. melanopterus* hybrid	blood (live bird), Bali Bird Park	Y	Pending
BBP10*	*A. m. melanopterus*	blood (live bird), Bali Bird Park	Y	Pending
BBP11	*A. m. melanopterus*	blood (live bird), Bali Bird Park	Y	Pending
BBP12	*A. m. melanopterus*	blood (live bird), Bali Bird Park	Y	Pending
BBP13*	*A. m. melanopterus* hybrid	blood (live bird), Bali Bird Park	Y	Pending
BWS 000	*A. m. melanopterus*	tissue (deceased bird), Cikananga Wildlife Center	N	Pending
BWS 008	*A. m. melanopterus* hybrid	blood (live bird), Bali Bird Park	Y	Pending
BWS 011	*A. m. melanopterus*	tissue (deceased bird), Cikananga Wildlife Center	N	Pending
BWS 012	*A. m. melanopterus*	tissue (deceased bird), Cikananga Wildlife Center	N	Pending
BWS 013	*A. m. melanopterus*	blood (live bird), Cikananga Wildlife Center	N	Pending
BWS 030	*A. m. melanopterus*	blood (live bird), Cikananga Wildlife Center	N	Pending
BWS 031	*A. m. melanopterus*	blood (live bird), Cikananga Wildlife Center	Y	Pending
BWS 034	*A. m. melanopterus*	blood (live bird), Cikananga Wildlife Center	Y	Pending
BWS 035	*A. m. melanopterus*	blood (live bird), Cikananga Wildlife Center	N	Pending
BWS 041	*A. m. melanopterus*	blood (live bird), Cikananga Wildlife Center	N	Pending
BWS 063*	*A. m. melanopterus*	blood (live bird), Cikananga Wildlife Center	N	Pending
BWS 079	*A. m. melanopterus*	blood (live bird), Cikananga Wildlife Center	Y	Pending
BWS 084	*A. m. melanopterus*	blood (live bird), Cikananga Wildlife Center	N	Pending
BWS 085	*A. m. melanopterus*	blood (live bird), Cikananga Wildlife Center	Y	Pending
BWS 094*	*A. m. melanopterus*	blood (live bird), Cikananga Wildlife Center	N	Pending
BWS 107	*A. m. melanopterus*	blood (live bird), Cikananga Wildlife Center	N	Pending
BWS 115	*A. m. melanopterus*	blood (live bird), Cikananga Wildlife Center	Y	Pending
BWS 155	*A. m. melanopterus*	blood (live bird), Cikananga Wildlife Center	Y	Pending
BWS 157	*A. m. melanopterus*	blood (live bird), Cikananga Wildlife Center	Y	Pending
BWS 190	*A. m. melanopterus*	tissue (deceased bird), Cikananga Wildlife Center	N	Pending
BWS 211	*A. m. melanopterus*	tissue (deceased bird), Cikananga Wildlife Center	N	Pending
BWS 356	*A. m. melanopterus*	blood (live bird), Cikananga Wildlife Center	N	Pending
BWS 358	*A. m. melanopterus*	blood (live bird), Cikananga Wildlife Center	Y	Pending
BWS 361	*A. m. melanopterus*	blood (live bird), Cikananga Wildlife Center	Y	Pending
BWS 362	*A. m. melanopterus*	tissue (deceased bird), Cikananga Wildlife Center	N	Pending
BWS 367	*A. m. melanopterus*	blood (live bird), Cikananga Wildlife Center	Y	Pending
BWS 381	*A. m. melanopterus*	tissue (deceased bird), Cikananga Wildlife Center	N	Pending
BWS 385	*A. m. melanopterus*	blood (live bird), Cikananga Wildlife Center	Y	Pending
BWS 388	*A. m. melanopterus*	tissue (deceased bird), Cikananga Wildlife Center	N	Pending
BWS 389	*A. m. melanopterus*	blood (live bird), Cikananga Wildlife Center	Y	Pending
BWS 393	*A. m. melanopterus*	blood (live bird), Cikananga Wildlife Center	N	Pending
BWS 395	*A. m. melanopterus*	blood (live bird), Cikananga Wildlife Center	Y	Pending
BWS 399	*A. m. melanopterus*	blood (live bird), Cikananga Wildlife Center	N	Pending
BWS 405	*A. m. melanopterus*	blood (live bird), Cikananga Wildlife Center	Y	Pending
BWS 406	*A. m. melanopterus*	tissue (deceased bird), Cikananga Wildlife Center	N	Pending
BWS 412	*A. m. melanopterus*	tissue (deceased bird), Cikananga Wildlife Center	N	Pending
BWS 423	*A. m. melanopterus*	tissue (deceased bird), Cikananga Wildlife Center	N	Pending
BWS 433	*A. m. melanopterus*	tissue (deceased bird), Cikananga Wildlife Center	N	Pending
BWS 443	*A. m. melanopterus*	tissue (deceased bird), Cikananga Wildlife Center	N	Pending
BWS 465	*A. m. melanopterus*	tissue (deceased bird), Cikananga Wildlife Center	N	Pending
BWS 472	*A. m. melanopterus*	tissue (deceased bird), Cikananga Wildlife Center	N	Pending
BWS 489	*A. m. melanopterus*	tissue (deceased bird), Cikananga Wildlife Center	N	Pending
BWS 491	*A. m. melanopterus*	tissue (deceased bird), Cikananga Wildlife Center	N	Pending
BWS 514	*A. m. melanopterus*	tissue (deceased bird), Cikananga Wildlife Center	N	Pending
BWS 519	*A. m. melanopterus*	blood (live bird), Cikananga Wildlife Center	Y	Pending
BWS 524	*A. m. melanopterus*	blood (live bird), Cikananga Wildlife Center	N	Pending
BWS 525	*A. m. melanopterus*	blood (live bird), Cikananga Wildlife Center	N	Pending
BWS 536	*A. m. melanopterus*	tissue (deceased bird), Cikananga Wildlife Center	N	Pending
BWS 537	*A. m. melanopterus*	tissue (deceased bird), Cikananga Wildlife Center	N	Pending
BWS 548	*A. m. melanopterus*	tissue (deceased bird), Cikananga Wildlife Center	N	Pending
BWS 549	*A. m. melanopterus*	tissue (deceased bird), Cikananga Wildlife Center	N	Pending
BWS 557	*A. m. melanopterus*	tissue (deceased bird), Cikananga Wildlife Center	N	Pending
BWS 559	*A. m. melanopterus*	tissue (deceased bird), Cikananga Wildlife Center	N	Pending
BWS 565	*A. m. melanopterus*	tissue (deceased bird), Cikananga Wildlife Center	N	Pending
BWS 566	*A. m. melanopterus*	blood (live bird), Cikananga Wildlife Center	N	Pending
BWS 568	*A. m. melanopterus*	tissue (deceased bird), Cikananga Wildlife Center	N	Pending
BWS 570	*A. m. melanopterus*	blood (live bird), Cikananga Wildlife Center	Y	Pending
BWS 571	*A. m. melanopterus*	blood (live bird), Cikananga Wildlife Center	N	Pending
BWS 572	*A. m. melanopterus*	blood (live bird), Cikananga Wildlife Center	Y	Pending
BWS 576	*A. m. melanopterus*	blood (live bird), Cikananga Wildlife Center	N	Pending
BWS 577˜	*A. m. melanopterus*	blood (live bird), Cikananga Wildlife Center	Y	Pending
BWS 592	*A. m. melanopterus*	blood (live bird), Cikananga Wildlife Center	N	Pending
BWS 593	*A. m. melanopterus*	blood (live bird), Cikananga Wildlife Center	Y	Pending
BWS 595	*A. m. melanopterus*	blood (live bird), Cikananga Wildlife Center	N	Pending
BWS 596	*A. m. melanopterus*	blood (live bird), Cikananga Wildlife Center	N	Pending
BWS 597	*A. m. melanopterus*	blood (live bird), Cikananga Wildlife Center	N	Pending
BWS 599	*A. m. melanopterus*	tissue (deceased bird), Cikananga Wildlife Center	N	Pending
BWS 605˜	*A. m. melanopterus*	blood (live bird), Cikananga Wildlife Center	N	Pending
BWS 606	*A. m. melanopterus*	blood (live bird), Cikananga Wildlife Center	N	Pending
BWS 607	*A. m. melanopterus*	tissue (deceased bird), Cikananga Wildlife Center	N	Pending
BWS 609	*A. m. melanopterus*	blood (live bird), Cikananga Wildlife Center	N	Pending
BWS 611	*A. m. melanopterus*	blood (live bird), Cikananga Wildlife Center	N	Pending
BWS 612	*A. m. melanopterus*	blood (live bird), Cikananga Wildlife Center	N	Pending
BWS 615	*A. m. melanopterus*	blood (live bird), Cikananga Wildlife Center	Y	Pending
BWS 616	*A. m. melanopterus*	tissue (deceased bird), Cikananga Wildlife Center	N	Pending
BWS 619	*A. m. melanopterus*	tissue (deceased bird), Cikananga Wildlife Center	N	Pending
BWS 620	*A. m. melanopterus* hybrid	blood (live bird), Cikananga Wildlife Center	Y	Pending
BWS 621	*A. m. melanopterus* hybrid	blood (live bird), Cikananga Wildlife Center	Y	Pending
BWS 622	*A. m. melanopterus*	blood (live bird), Cikananga Wildlife Center	Y	Pending
BWS 623	*A. m. melanopterus* hybrid	blood (live bird), Cikananga Wildlife Center	Y	Pending
BWS 624	*A. m. melanopterus*	blood (live bird), Cikananga Wildlife Center	N	Pending
BWS 625	*A. m. melanopterus*	blood (live bird), Cikananga Wildlife Center	Y	Pending
BWS 626	*A. m. melanopterus*	blood (live bird), Cikananga Wildlife Center	N	Pending
C24	*Acridotheres javanicus*	tissue (deceased bird), Singapore	N	Pending
C328	*Acridotheres javanicus*	tissue (deceased bird), Singapore	N	Pending
C349	*Acridotheres javanicus*	tissue (deceased bird), Singapore	N	Pending
C402	*Acridotheres javanicus*	tissue (deceased bird), Singapore	N	Pending
C505	*Acridotheres javanicus*	tissue (deceased bird), Singapore	N	Pending
C522	*Acridotheres javanicus*	tissue (deceased bird), Singapore	N	Pending
C69	*Acridotheres tristis*	tissue (deceased bird), Singapore	N	Pending
C662	*Acridotheres tristis*	tissue (deceased bird), Singapore	N	Pending
C689	*Acridotheres tristis*	tissue (deceased bird), Singapore	N	Pending

All samples were sourced from captivity or Genbank. Samples marked with an * and ˜ were additionally processed to obtain *ND2* and *MC1R* sequences respectively.

### Morphological scoring system

We photographed 39 live individuals from conservation breeding facilities for morphological scoring, spanning the full spectrum of morphological phenotypes from the lightest bird (presumably pure *melanopterus*; colour score range 0–6) to the darkest bird (presumably pure *tertius*; colour score 24; Fig. 2). Our cumulative scoring system was designed so as to evaluate the incidence of melanism or ‘greyness’ across multiple plumage regions. Birds that were not photographed were classified as ‘Unknown’.

A total of six morphological features were evaluated to score individuals along a melanism gradient. The six features are; (i) secondary coverts (comprising lesser, median and greater secondary coverts), (ii) scapulars, (iii) mantle, (iv) mid back, (v) rump, and (vi) tail coverts. Scores across all six features were then added up to produce a single aggregate colour score for each individual. All individuals were scored by a single researcher to preclude observer bias.

A K means cluster analysis using the R package Cluster^75^ was then conducted using the original data representing the percentage scores for each individual to determine if the analysed birds fall into distinct morphological clusters, and elucidate the optimum number of clusters suitable to describe the data.

### DNA extraction and single-locus sequencing

DNA extractions were carried out using the Qiagen DNEasy Blood & Tissue Kit following the manufacturer’s recommended protocol. A Qubit 2.0 Fluorometer was used to quantify DNA concentrations of the extracts. A subset of seven samples were selected for sequencing of the mitochondrial gene NADH dehydrogenase subunit 2 (*ND2*)^76^, including three *melanopterus*, one *tertius* and three presumed morphological hybrids, whilst a similarly diverse subset of five were selected for Melanocortin-1 receptor (*MC1R*)^77^ sequencing. The subsets of samples chosen for *ND2* and *MC1R* sequencing were selected to represent the entire breadth of taxonomic and melanistic variation within our larger dataset, respectively. Polymerase chain reaction (PCR) was carried out in a C1000 Thermal Cycler. We performed PCR amplifications in individual 25 μl reaction volumes, which comprised 2.5 μl DreamTaq buffer, 0.5 μl dNTP mix (working concentration 10 mM), 0.5 μl of each primer (working concentration 10 μm), 0.125 μl DreamTaq polymerase, 2 μl mtDNA template and 18.8 μl molecular grade water. PCR product clean-up was carried out using ExoSAP-IT, and the BigDye Terminator v3.1 Cycle Sequencing Kit (Applied Biosystems Inc.) was used to cycle-sequence the samples. Sequences were obtained by capillary electrophoresis using an Applied Biosystems 3130xl Genetic Analyzer.

### ddRADSeq library preparation

Double-digest restriction enzyme associated DNA sequencing (ddRADSeq) was performed as per Tang et al.^78^. Samples were split into total DNA yield bands of either 500 or 200 ng, based on their post-extraction concentrations, and input volumes for the first restriction step were calculated based on these cut-off values. Restriction enzymes EcoRI and MsP1 (New England Biolabs Inc.) were used to double digest the samples for 3.5hrs at 37 °C, followed by clean-up using a 1.1X ratio of Sera-Mag SpeedBead Carboxylate-Modified Magnetic Particles (Thermo Scientific). Samples were re-quantified and then ligated with unique barcodes (PIE adaptors) by T4 DNA Ligase (New England Biolabs Inc.) at 16 °C for 16 h.

For subsequent library preparation, the samples were split into eight pools for distribution across two Illumina HiSeq 4000 lanes (4 pools/lane) according to their post-restriction concentrations. Samples similar in concentration were pooled to avoid either over- or under-representation of any one sample. Library clean ups were performed for each of the eight pools with AMPure XP beads (Agencourt) using a 1.5X bead ratio. Size selection was carried out for these eight pools using a Pippin Prep Gel Electrophoresis system (Sage Science) to isolate fragments for a sample peak of 420 bp in length, followed by another AMPure XP clean up. PCR amplification of size-selected fragments was run for 12 cycles, followed by a final AMPure XP clean up step. Pools were screened for quality control on a Fragment Analyzer (Advanced Analytical) and quantified on a Qubit 2.0 Fluorometer before being pooled in equimolar proportions to form two final libraries. Final libraries were spiked with 30% PhiX and transferred to the Genome Institute of Singapore for Illumina sequencing, achieving a mean sequencing depth of 143X.

A subset of eleven Black-winged Myna (BWM) samples, including representatives of *melanopterus*, *tertius* and morphological hybrids, were additionally sequenced on a separate Illumina HiSeq 4000 lane to a mean sequencing depth of 359X to obtain more SNPs for introgression testing. We also sequenced three samples of Common Myna (*Acridotheres tristis*) and six samples of Javan Myna (*Acridotheres javanicus*) in the same way for introgression testing (Table 3).

### SNP calling for Next-generation sequencing data

We used FastQC (Babraham Bioinformatics) to analyze sequence quality across all base positions. Demultiplexing was then performed using the “process-radtags” command in Stacks v1.34^79^. Samples with less than one million reads were removed from subsequent analysis. The remaining sequence reads were then aligned against the Javan Myna (*Acridotheres javanicus*) reference genome^80^ using BWA-MEM^81^.

The pipeline ref_map.pl in Stacks v1.34^79^ was used to call SNPs. We employed a minimum stack depth of 10 in our analyses. We only retained loci that were found in more than 90% of individuals, using options available in the populations module of Stacks. As a first step to reduce the effects of linkage disequilibrium in subsequent analyses, we only accepted one SNP from each locus using the –write_single_snp option provided, leading to a set of 7,592 SNPs.

PLINK 2.0^82^ was run to determine the amount of missing data present across the 85 retained samples and filter out any individuals with more than 30% missing data, and filter SNPs under linkage disequilibrium (r^2 ^> 0.9) using a 25-SNP window sliding 10 SNPs at a time.

We used the R package SNPRelate^83^ to estimate pairwise relatedness by means of maximum likelihood estimation. We then removed a single individual from each pair which displayed a kinship coefficient above 0.25 (first order kinship). We generated a total of four datasets using the methods described above, with the exception of employing a stack depth of 5 rather than 10 in the ref_map.pl pipeline for dataset (4) in order to retain more loci (Table 1).

Dataset 1 was used for detection of kin, whilst population genetic analyses such as principal component analysis (PCA) were run using both datasets 2 and 3 for comparison. Dataset 4 was used for introgression testing and fineRADstructure, and all datasets were used for comparative STRUCTURE analyses.

### Population differentiation

We assessed population subdivision of BWMs using a model-based clustering approach implemented in STRUCTURE v2.3.4^84^ for all datasets. STRUCTURE was run from *K* = 2 to *K* = 10 with ten iterations per *K* and without a priori hypotheses of cluster membership. For each iteration we implemented a burn-in of 50 000 generations and Markov chain Monte Carlo iterations (MCMC) for 250,000 generations. We used STRUCTURE Harvester Web v0.6.94^85^ and the Evanno method^47^ to determine the statistically most significant *K* value. Results were averaged across replicates by evaluating individual ancestry coefficients (q values) with CLUMPP v1.1.2^86^ using the Greedy option provided. Results for dataset 2 (Fig. 2) and dataset 4 (Fig. S2) are shown. We also explored population structure in dataset 2 by means of principal component analysis (PCA) in the R package SNPRelate, using a genetic covariance matrix calculated from genotypes.

We investigated individual-based pairwise coancestry using the MCMC coalescence algorithm as implemented in fineRADstructure v0.3.1^65^ based on haplotype linkage information. For this analysis we used dataset 4 (Table 1), excluding Common Myna samples, in order to compare genetic similarity of the *melanopterus* and *tertius* samples to a well-established sympatric species (Javan Myna). We used a missing data cut-off of 10%, and utilized RADpainter to calculate the coancestry matrix followed by assigning individuals to populations at default parameters, including a burn-in period of 100 000 and 100 000 MCMC iterations.

### Introgression testing

We tested a hypothesis of genetic introgression between BWMs and the congeneric, sympatric Javan Myna with the classic ABBA-BABA test^54^ using SNP dataset 4. A simple version of this test uses the relative frequencies of two discordant SNP distributions (ABBA vs BABA) in a four-taxon pectinate phylogenetic topology (Fig. 4) to detect post-divergence hybridization between discrete populations or lineages. Both SNP distributions can be generated by incomplete lineage sorting under a null hypothesis of zero cross-lineage gene flow, but the occurrence of genetic introgression from a donor (P3, Fig. 4) into a recipient (P2, Fig. 4) would lead to a preponderance of ABBA-like SNPs over BABA-like SNPs that can be estimated with a single statistic, D, as per Green et al.^54^.

We used the Dsuite package^87^ to test the hypothesis of gene flow from Javan Mynas (P3) into *tertius* (P2) relative to *melanopterus* (P1), and vice versa, using Common Mynas as the outgroup (Fig. 4). This was first done at the population level with population allele frequencies; and subsequently at the individual level, by testing all pairwise combinations of *melanopterus* and *tertius* individuals, set as P1 and P2 respectively, while using population allele frequencies for Javan Myna and Common Myna populations for P3 and the outgroup respectively. Finally, we estimated the genome-wide fraction of introgressive admixture, *f*_G_, for *tertius* as per Green et al.^54^, for both the population level test and for all pairwise combinations showing significant introgression.

### *ND2* and *MC1R* analyses

DNA sequences from Sanger sequencing were vetted using CodonCode Aligner version 8.0.1 (CodonCode Corporation). We supplemented our ND2 dataset with additional Black-winged Myna sequences from GenBank, including two *tertius*, one sequence with no subspecific assignment and a single sequence of the intermediate taxon *tricolor*, which is unrepresented in our genomic sampling. A Hill Myna *Gracula religiosa* sequence was also included for outgroup rooting. Samples were aligned with MEGA version 7^88^ using the ClustalW algorithm^89^ for a final alignment of 976 base pairs (bp) length. We ran a maximum likelihood tree using RAxML GUI 1.5^90^ under a GTR + Gamma model of sequence evolution and a rapid bootstrap algorithm with 10 000 replicates. A trimmed alignment of 902 bp, which excluded any ambiguous positions or gaps, was used to generate a median-joining haplotype network using Network version 10^91^.

*MC1R* sequences from six individuals were assembled as described above in order to generate an 835 base pair alignment. Nucleotide sequences were translated into amino acids using MEGA version 7 and compared to check for non-silent mutations using Geneious 11.1.5 (https://www.geneious.com).

### Ethics statement

All experimental protocols were approved and conducted in accordance with regulations outlined by the National University of Singapore’s Office of Safety, Health, and Environment. Animal sampling was approved by the Institutional Animal Care and Use Committee (IACUC) for the National University of Singapore.

## Supplementary information

Supplementary Information.

## Data Availability

The Black-winged Myna genomic data are pending accession on GenBank NCBI. Raw Stacks output and custom scripts are available from the corresponding authors upon request. All other data analysed during this study are included in the Supplementary information files.

## References

[1] Wilson KA, McBride MF, Bode M, Possingham HP (2006). Prioritizing global conservation efforts. Nature.

[2] Barnosky AD, Matzke N, Tomiya S, Wogan GO, Swartz B, Quental TB, Marshall C, McGuire JL, Lindsey EL, Maguire KC (2011). Has the Earth’s sixth mass extinction already arrived?. Nature.

[3] Sangster G (2009). Increasing numbers of bird species result from taxonomic progress, not taxonomic inflation. Proc. R. Soc. B Biol. Sci..

[4] Garnett ST, Christidis L (2017). Taxonomy anarchy hampers conservation. Nat. News.

[5] Thomson SA, Pyle RL, Ahyong ST, Alonso-Zarazaga M, Ammirati J, Araya JF, Ascher JS, Audisio TL, Azevedo-Santos VM, Bailly N (2018). Taxonomy based on science is necessary for global conservation. PLoS Biol..

[6] Winter M, Devictor V, Schweiger O (2013). Phylogenetic diversity and nature conservation: where are we?. Trends. Ecol..

[7] Haig SM, Beever EA, Chambers SM, Draheim HM, Dugger BD, Dunham S, Elliott-Smith E, Fontaine JB, Kesler DC, Knaus BJ (2006). Taxonomic considerations in listing subspecies under the US Endangered Species Act. Conserv. Biol..

[8] Mace GM (2004). The role of taxonomy in species conservation. Phil. Trans. R. Soc. B.

[9] Faith DP (2008). Threatened species and the potential loss of phylogenetic diversity: conservation scenarios based on estimated extinction probabilities and phylogenetic risk analysis. Conserv. Biol..

[10] Baack EJ, Rieseberg LH (2007). A genomic view of introgression and hybrid speciation. Curr. Opin. Genet. Dev..

[11] Rheindt FE, Edwards SV (2011). Genetic introgression: an integral but neglected component of speciation in birds. Auk.

[12] Hedrick PW (2013). Adaptive introgression in animals: examples and comparison to new mutation and standing variation as sources of adaptive variation. Mol. Ecol..

[13] Racimo F, Sankararaman S, Nielsen R, Huerta-Sánchez E (2015). Evidence for archaic adaptive introgression in humans. Nat. Rev. Genet..

[14] Pardo-Diaz C, Salazar C, Baxter SW, Merot C, Figueiredo-Ready W, Joron M, McMillan WO, Jiggins CD (2012). Adaptive introgression across species boundaries in Heliconius butterflies. PLoS Genet..

[15] Keller I, Wagner CE, Greuter L, Mwaiko S, Selz OM, Sivasundar A, Wittwer S, Seehausen O (2013). Population genomic signatures of divergent adaptation, gene flow and hybrid speciation in the rapid radiation of Lake Victoria cichlid fishes. Mol. Ecol..

[16] Rheindt FE, Fujita MK, Wilton PR, Edwards SV (2014). Introgression and phenotypic assimilation in Zimmerius flycatchers (Tyrannidae): population genetic and phylogenetic inferences from genome-wide SNPs. Syst. Biol..

[17] Lamichhaney S, Berglund J, Almén MS, Maqbool K, Grabherr M, Martinez-Barrio A, Promerová M, Rubin CJ, Wang C, Zamani N (2015). Evolution of Darwin’s finches and their beaks revealed by genome sequencing. Nature.

[18] Toews DP, Brelsford A, Grossen C, Milá B, Irwin DE (2016). Genomic variation across the Yellow-rumped Warbler species complex. Auk.

[19] Ng NS, Wilton PR, Prawiradilaga DM, Tay YC, Indrawan M, Garg KM, Rheindt FE (2017). The effects of Pleistocene climate change on biotic differentiation in a montane songbird clade from Wallacea. Mol. Phylogenet. Evol..

[20] Stryjewski KF, Sorenson MD (2017). Mosaic genome evolution in a recent and rapid avian radiation. Nat. Ecol. Evol..

[21] Huerta-Sánchez E, Jin X, Bianba Z, Peter BM, Vinckenbosch N, Liang Y, Yi X, He M, Somel M, Ni P (2014). Altitude adaptation in Tibetans caused by introgression of Denisovan-like DNA. Nature.

[22] Vernot B, Akey JM (2014). Resurrecting surviving Neandertal lineages from modern human genomes. Science.

[23] Shepherd CR, Nijman V, Krishnasamy K, Eaton JA, Chng SC (2016). Illegal trade pushing the Critically Endangered Black-winged Myna Acridotheres melanopterus towards imminent extinction. Bird. Conserv. Int..

[24] Feare CJ, Craig A (1998). Starlings and Mynas.

[25] Eaton JA, Shepherd CR, Rheindt FE, Harris JBC, Van Balen S, Wilcove DS, Collar NJ (2015). Trade-driven extinctions and near-extinctions of avian taxa in Sundaic Indonesia. Forktail.

[26] Eaton JA, van Balen S, Brickle NW, Rheindt FE (2016). Birds of the Indonesian Archipelago: Greater Sundas and Wallacea.

[27] Craig, A., Feare, C., de Juana, E., Sharpe, C. J., & Christie, D. A. Black-winged Myna (*Acridotheres melanopterus*). In *Handbook of the Birds of the World Alive* (eds del Hoyo, J., et al.). https://www.hbw.com/node/60876 (2019).

[28] del Hoyo, J., Collar, N., & Christie, D. A. Grey-backed Myna (*Acridotheres tricolor*). In *Handbook of the Birds of the World Alive* (eds del Hoyo, J., et al.). https://www.hbw.com/node/1344003 (2019).

[29] del Hoyo, J., Collar, N., & Christie, D. A. Grey-rumped Myna (*Acridotheres tertius*). In *Handbook of the Birds of the World Alive* (eds del Hoyo, J., et al.). https://www.hbw.com/node/1344004 (2019).

[30] Collar NJ, Gardner L, Jeggo DF, Marcordes B, Owen A, Pagel T, Pes T, Vaidl A, Wilkinson R, Wirth R (2012). Conservation breeding and the most threatened birds in Asia. BirdingAsia.

[31] Owen A, Wilkinson R, Sözer R (2014). In situ conservation breeding and the role of zoological institutions and private breeders in the recovery of highly endangered Indonesian passerine birds. Int. Zoo Yearb..

[32] Lee, J. G. H., Chng, S. C. L., & Eaton, J. A. Conservation strategy for Southeast Asian songbirds in trade. In *Recommendations from the First Asian Songbird Trade Crisis Summit 2015 Held in Jurong Bird Park, Singapore, 27–29 September 2015* (2016).

[33] BirdLife International. The IUCN Red List of Threatened Species 2018: e.T103870843A131892465. 10.2305/IUCN.UK.2018-2.RLTS.T103870843A131892465.en (2018).

[34] Del Hoyo J, Collar NJ (2016). HBW and BirdLife International Illustrated Checklist of the Birds of the World.

[35] Christidis, L. et al. *The Howard and Moore Complete Checklist of the Birds of the World*, version 4.1 (Downloadable checklist). https://www.howardandmoore.org (2018).

[36] Clements, J. F., et al. *The eBird/Clements checklist of birds of the world: v2019*. https://www.birds.cornell.edu/clementschecklist/download/ (2018).

[37] Gill, F., & Donsker, D. (Eds). *IOC World Bird List (v9.1)*. https://www.worldbirdnames.org/ (2019).

[38] Harrisson KA, Pavlova A, Gonçalves da Silva A, Rose R, Bull JK, Lancaster ML, Murray N, Quin B, Menkhorst P, Magrath MJ, Sunnucks P (2016). Scope for genetic rescue of an endangered subspecies though re-establishing natural gene flow with another subspecies. Mol. Ecol..

[39] Baveja P, Tang Q, Lee JG, Rheindt FE (2019). Impact of genomic leakage on the conservation of the endangered Milky Stork. Biol. Conserv..

[40] Zink RM, Groth JG, Vázquez-Miranda H, Barrowclough GF (2013). Phylogeography of the California Gnatcatcher (*Polioptila californica*) using multilocus DNA sequences and ecological niche modeling: implications for conservation. Auk.

[41] Chattopadhyay B, Garg KM, Soo YJ, Low GW, Frechette JL, Rheindt FE (2019). Conservation genomics in the fight to help the recovery of the critically endangered Siamese crocodile *Crocodylus siamensis*. Mol. Ecol..

[42] Sadanandan KR, Küpper C, Low GW, Yao CT, Li Y, Xu T, Rheindt FE, Wu S (2019). Population divergence and gene flow in two East Asian shorebirds on the verge of speciation. Sci. Rep..

[43] Ng EY, Garg KM, Low GW, Chattopadhyay B, Oh RR, Lee JG, Rheindt FE (2017). Conservation genomics identifies impact of trade in a threatened songbird. Biol. Conserv..

[44] Nash HC, Low GW, Choo SW, Chong JL, Semiadi G, Hari R, Sulaiman MH, Turvey ST, Evans TA, Rheindt FE (2018). Conservation genomics reveals possible illegal trade routes and admixture across pangolin lineages in Southeast Asia. Conserv. Genet..

[45] Çilingir FG, Rheindt FE, Garg KM, Platt K, Platt SG, Bickford DP (2017). Conservation genomics of the endangered Burmese roofed turtle. Conserv. Biol..

[46] Çilingir FG, Seah A, Horne BD, Som S, Bickford DP, Rheindt FE (2019). Last exit before the brink: conservation genomics of the Cambodian population of the critically endangered southern river terrapin. Ecol. Evol..

[47] Evanno G, Regnaut S, Goudet J (2005). Detecting the number of clusters of individuals using the software STRUCTURE: a simulation study. Mol. Ecol..

[48] Hebert PD, Stoeckle MY, Zemlak TS, Francis CM (2004). Identification of birds through DNA barcodes. PLoS Biol..

[49] Kerr KC, Stoeckle MY, Dove CJ, Weigt LA, Francis CM, Hebert PD (2007). Comprehensive DNA barcode coverage of North American birds. Mol. Ecol. Notes.

[50] Tizard J, Patel S, Waugh J, Tavares E, Bergmann T, Gill B, Norman J, Christidis L, Scofield P, Haddrath O (2019). DNA barcoding a unique avifauna: an important tool for evolution, systematics and conservation. BMC Evol. Biol..

[51] Shepherd CR, Cassey P (2017). Songbird trade crisis in Southeast Asia leads to the formation of IUCN SSC Asian Songbird Trade Specialist Group. J. Indo. Nat. Hist..

[52] Nijman V, Langgeng A, Birot H, Imron MA, Nekaris KAI (2018). Wildlife trade, captive breeding and the imminent extinction of a songbird. Glob. Ecol. Conserv..

[53] Symes WS, Edwards DP, Miettinen J, Rheindt FE, Carrasco LR (2018). Combined impacts of deforestation and wildlife trade on tropical biodiversity are severely underestimated. Nat. Commun..

[54] Green RE, Krause J, Briggs AW, Maricic T, Stenzel U, Kircher M, Patterson N, Li H, Zhai W, Fritz MH (2010). A draft sequence of the Neandertal genome. Science.

[55] Livneh B, Rajagopalan B (2017). Development of a gridded meteorological dataset over Java island, Indonesia 1985–2014. Sci. Data.

[56] Craig AJFK, Feare CJ, del Hoyo J (2009). Family Sturnidae (starlings). Handbook of the Birds of the World. Bush-Shrikes to Old World Sparrows.

[57] Zuccon D, Pasquet E, Ericson PG (2008). Phylogenetic relationships among Palearctic-Oriental starlings and mynas (genera Sturnus and Acridotheres: Sturnidae). Zool. Scr..

[58] Rheindt FE, Christidis L, Norman JA (2009). Genetic introgression, incomplete lineage sorting and faulty taxonomy create multiple cases of polyphyly in a montane clade of tyrant-flycatchers (Elaenia, Tyrannidae). Zool. Scr..

[59] Battey CJ, Klicka J (2017). Cryptic speciation and gene flow in a migratory songbird species complex: Insights from the red-eyed vireo (*Vireo olivaceus*). Mol. Phylogenet. Evol..

[60] Harris RB, Alström P, Ödeen A, Leaché AD (2018). Discordance between genomic divergence and phenotypic variation in a rapidly evolving avian genus (Motacilla). Mol. Phylogenet. Evol..

[61] Garg KM, Sam K, Chattopadhyay B, Sadanandan KR, Koane B, Ericson PG, Rheindt FE (2019). Gene flow in the Müllerian mimicry ring of a poisonous Papuan songbird clade (Pitohui; Aves). Genome Biol Evol.

[62] Zhang D, Tang L, Cheng Y, Hao Y, Xiong Y, Song G, Qu Y, Rheindt FE, Alström P, Jia C (2019). “Ghost Introgression” as a cause of deep mitochondrial divergence in a bird species complex. Mol. Biol. Evol..

[63] Uy JAC, Moyle RG, Filardi CE, Cheviron ZA (2009). Difference in plumage color used in species recognition between incipient species is linked to a single amino acid substitution in the melanocortin-1 receptor. Am. Nat..

[64] Janssen K, Mundy NI (2013). Molecular population genetics of the melanic plumage polymorphism in Arctic skuas (*Stercorarius parasiticus*): evidence for divergent selection on plumage colour. Mol. Ecol..

[65] Malinsky M, Trucchi E, Lawson DJ, Falush D (2018). RADpainter and fineRADstructure: population inference from RADseq data. Mol. Biol. Evol..

[66] Mech, L. D., & Rausch, R. A. The status of the wolf in the United States, 1973. IUCN Publications New Series Supplementary Paper No 43, 5 (1973).

[67] von Holdt BM, Pollinger JP, Earl DA, Knowles JC, Boyko AR, Parker H, Geffen E, Pilot M, Jedrzejewski W, Jedrzejewska B (2011). A genome-wide perspective on the evolutionary history of enigmatic wolf-like canids. Genome Res..

[68] Frueh, S. Current Evidence Supports Classification of Red Wolf as a Distinct Species, Report Says; Mexican Gray Wolf Is a Valid Subspecies of Gray Wolf. *The National Academies.* https://www8.nationalacademies.org/onpinews/newsitem.aspx?RecordID=25351 (2019).

[69] Antoniazza S, Burri R, Fumagalli L, Goudet J, Roulin A (2010). Local adaptation maintains clinal variation in melanin-based coloration of European Barn Owls (*Tyto alba*). Evolution.

[70] Irwin DE (2012). Local adaptation along smooth ecological gradients causes phylogeographic breaks and phenotypic clustering. Am. Nat..

[71] Harrisson KA, Amish SJ, Pavlova A, Narum SR, Telonis-Scott M, Rourke ML, Lyon J, Tonkin Z, Gilligan DM, Ingram BA (2017). Signatures of polygenic adaptation associated with climate across the range of a threatened fish species with high genetic connectivity. Mol. Ecol..

[72] Flanagan SP, Forester BR, Latch EK, Aitken SN, Hoban S (2018). Guidelines for planning genomic assessment and monitoring of locally adaptive variation to inform species conservation. Evol. Appl..

[73] Frankham R, Ballou JD, Ralls K, Eldridge M, Dudash MR, Fenster CB, Lacy RC, Sunnucks P (2017). Genetic Management of Fragmented Animal and Plant Populations.

[74] Voris HK (2000). Maps of Pleistocene sea levels in Southeast Asia: shorelines, river systems and time durations. J. Biogeogr..

[75] Maechler, M., Rousseeuw, P., Struyf, A., Hubert, M., Hornik, K., Studer, M., & Roudier, P. “Finding groups in data”: Cluster analysis extended. https://cran.r-project.org/web/packages/ cluster/index.html (2015).

[76] Sorenson MD, Ast JC, Dimcheff DE, Yuri T, Mindell DP (1999). Primers for a PCR-based approach to mitochondrial genome sequencing in birds and other vertebrates. Mol. Phylogenet. Evol..

[77] Cheviron ZA, Hackett SJ, Brumfield RT (2006). Sequence variation in the coding region of the melanocortin-1 receptor gene (*MC1R*) is not associated with plumage variation in the blue-crowned manakin (Lepidothrix coronata). Porc. R. Soc. B Biol. Sci..

[78] Tang Q, Low GW, Lim JY, Gwee CY, Rheindt FE (2018). Human activities and landscape features interact to closely define the distribution and dispersal of an urban commensal. Evol. Appl..

[79] Catchen J, Hohenlohe PA, Bassham S, Amores A, Cresko WA (2013). Stacks: an analysis tool set for population genomics. Mol. Ecol..

[80] Low GW, Chattopadhyay B, Garg KM, Irestedt M, Ericson PG, Yap G, Tang Q, Wu S, Rheindt FE (2018). Urban landscape genomics identifies fine-scale gene flow patterns in an avian invasive. Heredity.

[81] Li H, Durbin R (2009). Fast and accurate short read alignment with Burrows–Wheeler transform. Bioinformatics.

[82] Chang CC, Chow CC, Tellier LC, Vattikuti S, Purcell SM, Lee JJ (2015). Second-generation PLINK: rising to the challenge of larger and richer datasets. Gigascience.

[83] Zheng X, Levine D, Shen J, Gogarten SM, Laurie C, Weir BS (2012). A high-performance computing toolset for relatedness and principal component analysis of SNP data. Bioinformatics.

[84] Pritchard JK, Stephens M, Donnelly P (2000). Inference of population structure using multilocus genotype data. Genetics.

[85] Earl DA (2012). STRUCTURE HARVESTER: a website and program for visualizing STRUCTURE output and implementing the Evanno method. Conserv. Genet. Resour..

[86] Jakobsson M, Rosenberg NA (2007). CLUMPP: a cluster matching and permutation program for dealing with label switching and multimodality in analysis of population structure. Bioinformatics.

[87] Malinsky, M. Dsuite-fast D-statistics and related admixture evidence from VCF files. *BioRxiv* **634477** (2019). 10.1111/1755-0998.13265 PMC7116594 33012121

[88] Kumar S, Stecher G, Tamura K (2016). MEGA7: molecular evolutionary genetics analysis version 7.0 for bigger datasets. Mol. Biol. Evol..

[89] Thompson JD, Gibson TJ, Higgins DG (2003). Multiple sequence alignment using ClustalW and ClustalX. Curr. Protoc. Bioinform..

[90] Silvestro D, Michalak I (2012). raxmlGUI: a graphical front-end for RAxML. Org. Divers. Evol..

[91] Bandelt HJ, Forster P, Röhl A (1999). Median-joining networks for inferring intraspecific phylogenies. Mol. Biol. Evol..

