# Supplementary material for: The conservation value of admixed phenotypes in a critically endangered species complex
Source: Sci Rep. 2020 Sep 23;10:15549. doi: 10.1038/s41598-020-72428-2 (PMC7511927; doi:10.1038/s41598-020-72428-2)
Supplement: Supplementary file 1 — Supplementary Information. [file 41598_2020_72428_MOESM1_ESM.docx]

The Conservation Value of Admixed Phenotypes in a Critically Endangered Species Complex

**Keren R. Sadanandan, Gabriel W. Low, Sheeraja Sridharan, Chyi Yin Gwee, Elize Y. X. Ng, Pramana Yuda, Dewi M. Prawiradilaga, Jessica G. H. Lee, Anaïs Tritto, Frank E. Rheindt**


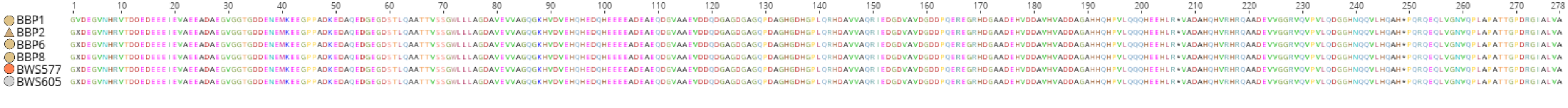


**Figure S1.** Translational alignment of *MC1R* gene sequence across a subset of Black-winged Myna samples, showing no non-synonymous mutations or sequence diversity.


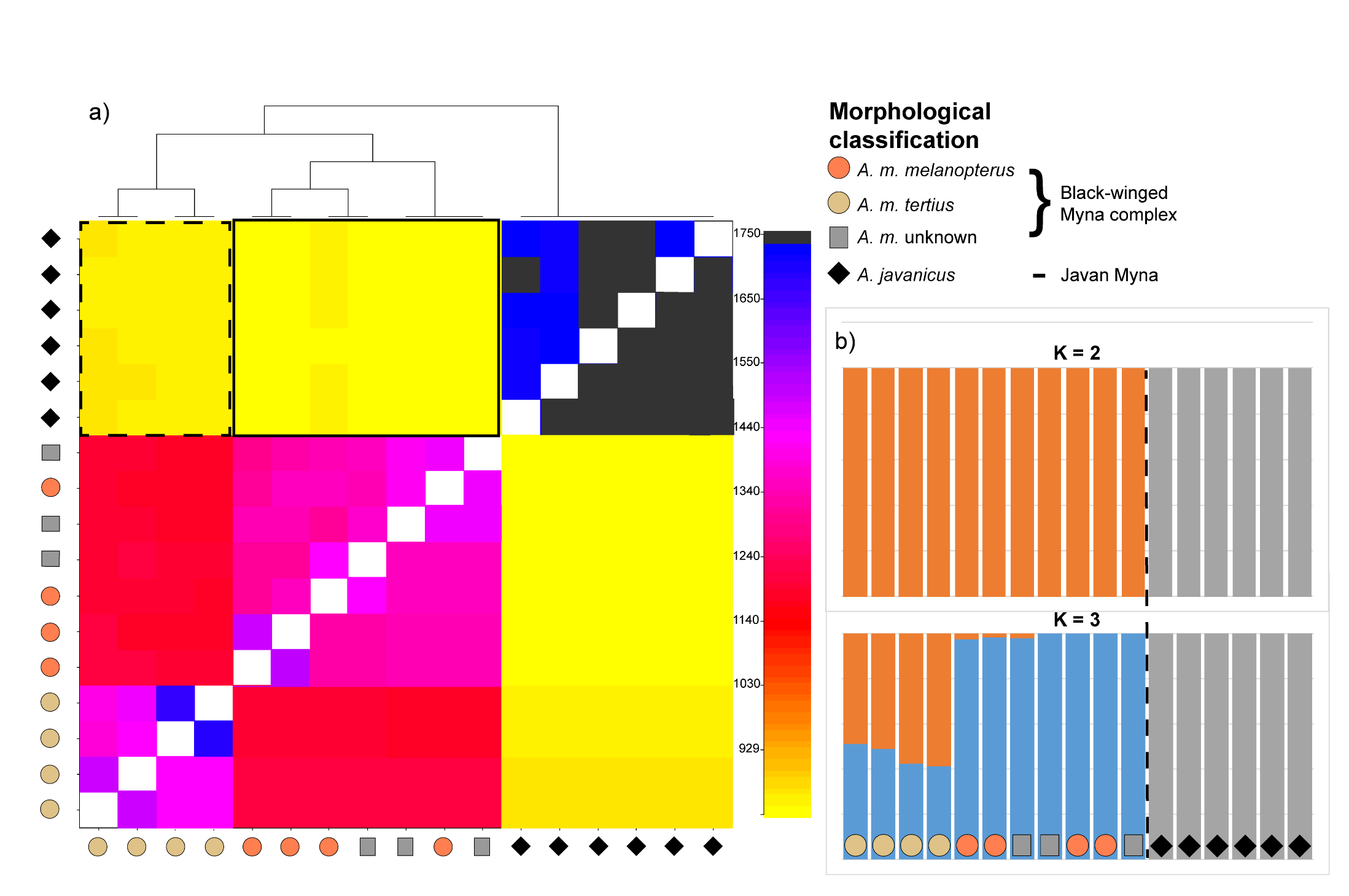


**Figure S2.** Patterns of shared allelic variation among Black-winged and Javan Mynas. a) Coancestry coefficients between individual Myna samples are plotted above the diagonal, and averaged coefficients between and among taxa or populations are plotted below. Higher coancestry values for each pairwise comparison are indicated by darker colors (see color spectrum on the right). The morphological identity of samples is indicated along axes. ‘*A. m.* unknown’ samples can be assumed to be of the *melanopterus* subspecies (see Materials and Methods section 2.2). The tree above the matrix shows relationships among all taxa compared and was generated using fineRADstructure’s algorithm. Stippled box highlights coancestry values between Javan Mynas and BWM subspecies A. m. tertius (darker yellow) and unbroken box highlights coancestry values between Javan Mynas and BWM subspecies *A. m. melanopterus* (lighter yellow). b) STRUCTURE results for K=2 and K=3 based on dataset 4 (Table S2; excluding Common Myna), showing genomic division between Javan Mynas and the two terminal BWM subspecies (*melanopterus* and *tertius*).
